# Investigating the Phase Transition Kinetics of 1-Octadecanol/Sorbitol Derivative/Expanded Graphite Composite Phase Change Material with Isoconversional and Multivariate Non-Linear Regression Methods

**DOI:** 10.3390/ma16217024

**Published:** 2023-11-03

**Authors:** Jun Xu, Yuanyuan Li, Xiaomin Cheng

**Affiliations:** 1 School of Materials Science and Engineering, Wuhan University of Technology, Wuhan 470070, China; xujun22@whut.edu.cn (J.X.); yyli@whut.edu.cn (Y.L.); 2 School of Electromechanical and Intelligent Manufacturing, Huanggang Normal University, Huanggang 438000, China

**Keywords:** organic composite phase change materials, differential scanning calorimetry, phase transition processes, non-isothermal kinetics, kinetic predictions

## Abstract

Organic composite phase change materials (PCMs) have been extensively studied, and it is important to investigate the effect of added components on the phase change process of the organic matrix. Herein, the phase transition process of the composite PCM with 1-octadecanol (OD) as the matrix adsorbed by a network framework composed of 1,3:2,4-di-(3,4-dimethyl) benzylidene sorbitol (DMDBS) and expanded graphite (EG) was measured using differential scanning calorimetry (DSC) at several linear heating rates. Using isoconversional and multivariate non-linear regression methods, a two-step consecutive reaction model for the composite PCM was established, while the apparent activation energies and pre-exponential factors were determined. The reaction mechanism of the first step was altered compared to pure OD, while the activation energies significantly decreased at the initial stage of the phase transition process and increased at the later stage. Combined with microscopic morphology analysis, the main reasons were the size and nanoconfinement effect. The predictions of the composite PCM under various conditions suggested that the composite PCM had a wider available temperature range compared to pure OD. This research provided a new idea for the in-depth study of the phase transition process of organic composite PCMs, which was helpful for the evaluation of organic composite PCMs.

## 1. Introduction

Various renewable energy sources, represented by solar energy, are volatile and intermittent [1]. Solar energy is affected by climatic conditions and hours of sunshine. Tidal and wind energy are also affected by climatic conditions. Wind energy, in particular, is relatively unpredictable. In addition, more than half of global energy is lost in the form of waste heat [2]. If this renewable energy and waste heat could be stored and reused when needed, it would significantly mitigate global climate deterioration. Using the latent heat storage properties of phase change materials (PCMs) can significantly increase the efficiency of energy storage [3,4]. Benefiting from their relatively stable properties and low price, PCMs are widely used in the fields of solar energy utilization, industrial waste heat recovery, building energy conservation, and the thermal protection of electronic devices [5,6,7,8]. Since phase change materials have a beneficial thermal energy storage capacity only in the phase transition temperature range, a wide range of PCMs with different melting points are required to cope with different scenarios.

At low to medium temperatures, the selected organic PCMs have good enthalpies of fusion, repeatable melting and solidification, small volume changes during phase transitions, corrosion resistance, and low cost [9,10]. Examples include alkanes (kinds of paraffin), alcohols, fatty acids, esters, etc., which have been extensively investigated and have great potential for thermal energy storage (TES) [11,12]. However, organic PCMs generally suffer from low thermal conductivity, susceptibility to leakage, and low photothermal conversion efficiency, which limit their wider application. To overcome the issues, different modification methods are designed and invented [13,14,15]. Graphite derivatives [16], carbon nanotubes (CNTs) [17], and carbon nanofibers (CNFs) [18] have high thermal conductivity and specific surface area, which can improve the thermal conductivity of organic PCMs while providing some mitigation of leakage in organic PCMs. Unfortunately, while they are effective in improving the thermal conductivity and photothermal conversion efficiency of composite PCMs, this single type of additive is not ideal for the encapsulation of organic PCMs.

Currently, there is a significant increase in research on shape-stabilized organic composite PCMs with various excellent properties, such as high thermal conductivity and good photothermal performance [19,20,21]. Serrano et al. [22] used solid–solid phase transition Neopentyl Glycol (NPG) as a supporting matrix for Docosane, up to 60% of docosane was adsorbed by decreasing the NPG crystal size and adding expanded graphite, which significantly improved the shape stability and thermal conductivity of the organic PCMs. Tan et al. [23] prepared a photothermal phase change hydrogel with high latent heat, reliable thermal stability, and good photothermal performance through sodium alginate (SA) hydrogel composite photothermal materials (CuS-CNTs) and pure polyethylene glycol 6000 (PEG-6000). Kook et al. [24] investigated the influence of synthesis conditions on the thermal properties and encapsulation effect of Polyurea nano-encapsulated phase change materials (PUA-NEPCMs). They found that increasing the PSSMA content enhanced the heat transfer properties of composite PCMs. Yuan et al. [25] designed a microcapsule encapsulating paraffin with a graphene oxide-modified mixture of ethyl and methyl cellulose as the wall material, which had good thermal reliability, high thermal storage capacity, and better photothermal conversion properties.

It is worth noting that, with the introduction of these modified materials, the phase transition process of organic PCMs tends to change as well. Liang et al. [26] prepared a shape-stabilized composite PCM based on lauric acid (LA) and graphene/graphene oxide aerogel, which had excellent energy storage and conductivity properties. The phase transition process of the composite PCM was investigated using crystallization kinetics, and the results showed that the aerogel skeleton had a heterogeneous nucleation effect on the crystallization of LA, which increased the crystallization rate of LA under non-isothermal and isothermal conditions. Song et al. [27] prepared a new shape-stable composite phase change material using the optimized expanded vermiculite (EVM) as a support material. The non-isothermal crystallization kinetics showed that the optimized EVM could effectively reduce the activation energy of nucleation and improve the crystallization and nucleation process of PEG. However, it also limited the crystal growth process of PEG. Most crystallization kinetics analyses are performed using means of single-step Avrami–Arrhenius treatment, but some of them, for complex phase transition processes, ignore the dependence of the activation energy on the conversion degree, making the results problematic [28]. Moreover, the crystallization kinetics analyses are relatively difficult for determining kinetic models for overlapping complex phase transition processes [29].

Non-isothermal kinetics, as the core of thermal analysis kinetics, has been widely applied to the kinetics of various endothermic reaction processes in inorganic [30], organic [31,32], and polymeric materials [33,34,35], as well as biomass [36] and solid fuels [37]. Of these, the multivariate non-linear regression method is suitable for analyzing strongly overlapping steps and complex processes where the reaction mechanism changes at different conversion degrees [29]. This approach has been applied to the study of the phase change behavior of organic PCMs [38,39], and more theoretical investigations are still needed.

Previously, we [40] selected a sorbitol derivative, 1,3:2,4-di-(3,4-dimethyl) benzylidene sorbitol (DMDBS), as the support material. DMDBS is a low-molecular-weight gelator, which can self-assemble to produce a one-dimensional supramolecular structure [41], forming a gel network to encapsulate the PCM. Compared with currently used encapsulation materials, such as microcapsules, porous materials, and polymer matrix, it can achieve an excellent encapsulation effect with less addition. A gelatinous shape-stabilized composite PCM was prepared with 1-octadecanol (OD) adsorbed by the three-dimensional network framework consisting of DMDBS and EG together. With the content of DMDBS and EG at only 3% and 6%, respectively, the composite PCM had a leakage of 4.86%, which reached a thermal conductivity of 4.276 W/m K, as well as maintaining a high enthalpy of phase transition and good thermal stability. These caused the composite PCMs to have great potential in specific fields requiring thermal management. At the same time, with the introduction of the modified materials, the phase transition process, especially the melting process, changed significantly compared to pure OD.

In this paper, the melting process of the OD/DMDBS/EG composite PCM at several linear heating rates was investigated using isoconversional and multivariate non-linear regression methods in non-isothermal kinetics. The kinetic triplet (the apparent activation energy, the pre-exponential factor, and the most appropriate kinetic model) of 1-octadecanol-based composite PCMs were determined for the first time. Much of the experimental data for each step of the complex phase transition process was reduced to a kinetic model with few parameters. Then, the mechanism of the phase transition process change was investigated in conjunction with the analysis of the microscopic morphology, and the kinetic parameters were corroborated in a physical sense. Finally, the phase transition processes of the composite PCM under various isothermal and non-isothermal conditions were predicted based on the obtained kinetic parameters. A convenient method for evaluating the available temperature range was provided for the practical application of composite PCMs, and theoretical guidance was provided for the design of new high-efficiency composite PCMs.

## 2. Materials and Methods

### 2.1. Materials

1,3:2,4-di-(3,4-dimethyl) benzylidene sorbitol (C24H30O6, DMDBS), 1-Octadecanol (C18H38O, OD), and n-hexane were obtained from Sinopharm Chemical Reagent Co., Ltd., Shanghai, China. Expandable graphite (50 meshes) was commercially supplied by Jinrilai Graphite Co., Ltd., Qingdao, China. All chemicals were used directly without further treatment.

### 2.2. Preparation

Expanded graphite (EG) was prepared by heating expandable graphite in a resistance furnace at 900 °C for 1 min and cooling at room temperature. It was then stored dry. A quantity of DMDBS was added to melted 1-octadecanol and heated with stirring until the DMDBS was fully melted. EG was added to the solution and stirring was continued until the solution was sufficiently adsorbed. Subsequently, the mixture was cooled naturally and held under a vacuum at 90 °C for 8 h to obtain OD/DMDBS/EG composite PCM. The mass ratio of the studied OD/DMDBS/EG composite PCM is 91:3:6 (wt.%) [40].

### 2.3. Characterization

The microstructure of the samples was observed using a Field Emission Scanning Electron Microscope (FE-SEM, S-4800, Hitachi, Tokyo, Japan) at 5 kV. The chemical structure of the samples was characterized with a Fourier Transform Infrared (FTIR) Spectrometer (Nicolet 6700, Laona, WI, USA) at a frequency range of 4000–400 cm^−1^.

A differential scanning calorimeter (DSC, NETZSCH 3500, Waldkraiburg, Germany), calibrated with indium and zinc standards, was used for the study of the melting process of the composite PCM. The samples weighed 5 ± 0.2 mg and were sealed in aluminum pans. All samples were first heated at a heating rate of 10 °C/min to 90 °C (the melting point of pure OD is 58.3 °C) for 10 min and then cooled at the same rate to 0 °C to start the test. All tests were carried out under a nitrogen atmosphere (50 mL/min). The endothermic phase transition process of the samples was tested at five rates of temperature increase, in the temperature range of 0 °C to 90 °C.

### 2.4. Kinetic Methods

In this study, the phase transition process of the samples is virtually massless and is a physical reaction involving an enthalpy change, and the conversion degree of phase transition α at a given temperature (or a given time) can be defined as in Equation (1):

(1) $$\alpha_{T}=\frac{1}{\Delta H}\int_{T_{0}}^{T}\left(\frac{dH}{dT}\right)dT$$

where ΔH denotes the total enthalpy of the phase transition process. ΔH was determined from the DSC endothermic curves and calculated using the DSC analysis software (NETZSCH Proteus Thermal Analysis Version 7.1.0). ΔH is proportional to peak area: ΔH = K·S, where K is an instrumental constant independent of temperature, and S is the peak area (integral of heat flow over time) determined for the heat absorption peak and baseline.

For constant heating rate non-isothermal conditions, the kinetics of the phase transition process can be described using Equation (2):

(2) $$\beta \frac{d\alpha }{dT}=A\exp\left(-\frac{E}{RT}\right)ƒ\left(\alpha \right)$$

where T denotes the absolute temperature (K), β denotes the linear heating rate (°C/min), and R denotes the general gas constant (J/mol K). A, E, and ƒ(α) are the kinetic triplet, denoting the pre-exponential factor (min^−1^), the apparent activation energy (J/mol), and the differential function of the reaction mechanism, respectively.

The corresponding integral form can be described using Equation (3):

(3) $$G\left(\alpha \right)=\frac{A}{\beta }\left(T-T_{0}\right)\exp\left(-\frac{E}{RT}\right)$$

where G(α) is the integral function of the reaction mechanism, ƒ(α) = 1/G′(α). T_0_ denotes the onset temperature at which the DSC curve deviates from the baseline. For linear heating conditions, T = T_0_ + βt.

In general, for non-isothermal kinetics, the dependence of apparent activation energy and conversion degree is first determined using a model-free isoconversional method before proceeding to the kinetic analysis that follows [42]. There were three model-free methods used in this study.

The Kissinger method calculates the data at the peak temperature (maximum conversion rate), and is based on equation [43]:

(4) $$\ln\left(\frac{\beta }{T_{P}{}^{2}}\right)=\ln\frac{AR}{E}-\frac{E}{RT_{P}}$$

where T_p_ is the peak temperature in thermal curves. The apparent activation energy was obtained by linear fitting the slope according to the plot ln(β/T_p_^2^) versus 1/T_p_.

The Friedman method is one of the most commonly used differential isoconversional conversion methods. Its equation is derived from the transformed form of Equation (2): (β/A)(dα/dT) = exp(−E/RT) ƒ(α). The left and right sides of this equation are dimensionless quantities. To facilitate the calculation of the kinetic parameters, both sides of the equation are taken logarithmically and converted. The Friedman method can be described by [44]:

(5) $$\ln\left(\frac{\beta d\alpha }{dT}\right)=\ln\left[Aƒ\left(\alpha \right)\right]-\frac{E}{RT}$$

For each given conversion degree, the apparent activation can be determined from the slope plot of ln [β(dα/dT)] against 1/T.

For the integral Ozawa–Flynn–Wall (OFW) method, the equation is derived from the type I kinetic integral equation for thermal analysis: G(α) = (AE/βR) P(μ), where P(μ) denotes the temperature integral, $P\left(\mu \right)=\int_{\infty }^{\mu }\left(-\frac{e^{-\mu }}{\mu^{2}}\right)d\mu$, and μ = E/RT. The Doyle approximation is used here: P(μ) = 0.00484e^−1.0516μ^, which is brought into the previous equation to obtain the equation G(α) = (AE/βR) 0.00484e^−1.0516μ^. The left and right sides of this equation are also dimensionless quantities. To facilitate the calculation of the kinetic parameters, both sides of the equation are taken logarithmically and converted. The Ozawa–Flynn–Wall (OFW) method can be defined as an equation [45]:

(6) $$\ln\beta =\ln\left[\frac{AE}{RG\left(\alpha \right)}\right]-5.331-1.052\left(\frac{E}{RT}\right)$$

The apparent activation can be determined from the slope plot of lnβ against 1/T.

The data of all samples calculated with the isoconversional methods were subsequently analyzed. If the apparent activation energy depends on the conversion degree, it should be fitted using multiple non-linear regression to determine the model of reaction mechanisms and the pre-exponential factor [46]. In this work, kinetic parameters were obtained though calculations with NETZSCH Thermokinetics 3.1 software [47]. The software includes a variety of reaction mechanism equations expanded on the types of expected response models given in the ICTAC recommendations [42]. Several reaction schemes are also given in the software. These include single-step, continuous, parallel, and reversible processes.

The results calculated with the isoconversional methods were used as initial conditions for multiple non-linear regression, brought into a lot of process reaction models combined with different mechanistic equations and reaction schemes, and then fitted using the thermokinetics software. Various results were evaluated using statistical techniques (e.g., least squares, correlation coefficients, F tests, etc.) to obtain the most statistically probable reaction model. Subsequently, based on the obtained kinetic parameters, the thermokinetics software predicted the phase transition processes of the composite PCM under isothermal and non-isothermal conditions, and some of the data were compared with the experimental results not involved in the calculations to test the most appropriate kinetic reaction model.

## 3. Results and Discussion

### 3.1. Thermal Analysis

**Figure 1:**
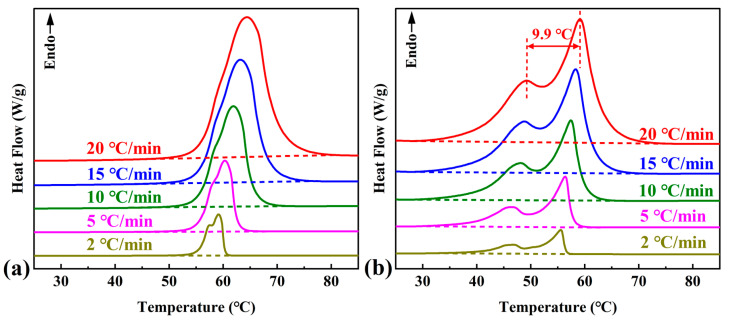
Differential scanning calorimeter (DSC) endothermic curves recorded at various linear heating rates for (**a**) pure OD and (**b**) OD/DMDBS/EG composite phase change material (PCM); the dashed lines are the baselines corresponding to each curve.

The DSC endothermic curves for pure OD and the investigated composite PCM at five linear heating rates are shown in Figure 1a,b. The peak temperatures of the melting peaks of the pure OD and the composite PCM were 62.1 °C and 57.2 °C, respectively, and the corresponding melting enthalpies were 246.1 J/g and 197.3 J/g, respectively (for a linear heating rate of 10 °C/min). The composite PCM still maintained a high enthalpy of phase change as a low-temperature phase change heat storage material. The detailed peak temperatures and melting enthalpies of the melting peaks at several linear heating rates are shown in Table 1.

As shown in Figure 1a,b, the peak temperature of endothermic peaks moved to higher temperatures as the heating rate increased, which could be due to the heat transfer hysteresis caused by the increase in the heating rate. When the heating rate was higher, the endothermic peaks of OD were close to a single peak, and with the decrease in the heating rate, the endothermic peaks showed a trend of peak separation. The main reason was that OD first transforms from the γ-phase (monoclinic) crystal structure to the rotamer phase (α-phase) during the melting process. During further heating, the α-phase melts into a liquid state. During the faster melting process, the solid–solid (γ-α) and solid–liquid (α-liquid) phase transitions occur very close to each other (within 1–2 °C). They are manifested as a single endothermic peak [48]. When the heating rate was lower, the two-phase transition no longer occurred in close proximity. For the composite PCM, the addition of DMDBS and EG caused the endothermic peaks to show a more pronounced peak separation (peak temperature difference of 9.9 °C for a linear heating rate of 20 °C/min), with endothermic peaks of the solid–solid phase transition shifting to lower temperatures.

### 3.2. Non-Isothermal Kinetic Analysis

#### 3.2.1. Isoconversional Method

**Figure 2:**
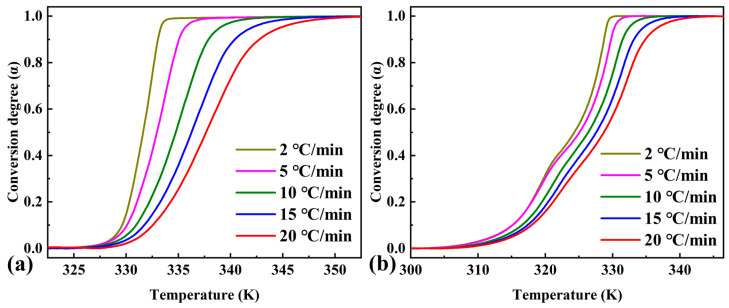
Dependence of conversion degree on temperature at various linear heating rates for (**a**) pure OD and (**b**) OD/DMDBS/EG composite.

**Figure 3:**
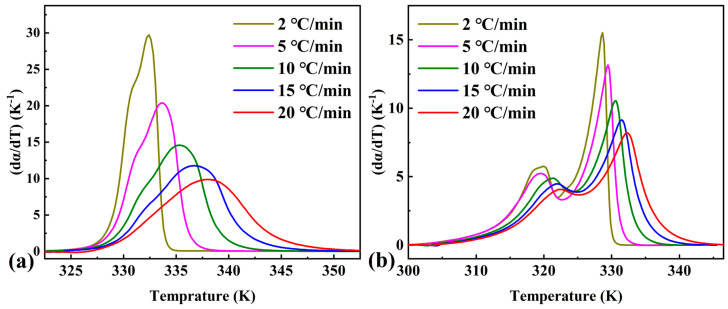
Dependence of conversion rate on temperature at various linear heating rates for (**a**) pure OD and (**b**) OD/DMDBS/EG composite.

From the endothermic curves for pure OD and the composite PCM, using Equation (1), the curves related to the conversion degree of phase transition (α) and temperature were calculated (Figure 2). Figure 3 shows the dependence of the conversion rate on the temperature calculated from Figure 2. As shown in Figure 3a,b, the conversion degree peaks of pure OD and the composite PCMs shifted to higher temperatures as the heating rate increased. The conversion rate versus temperature curves of the composite PCMs showed two conversion rate peaks corresponding to two endothermic peaks of the DSC curves. These data were then used to calculate the activation energy as a function of conversion degree.

**Figure 4:**
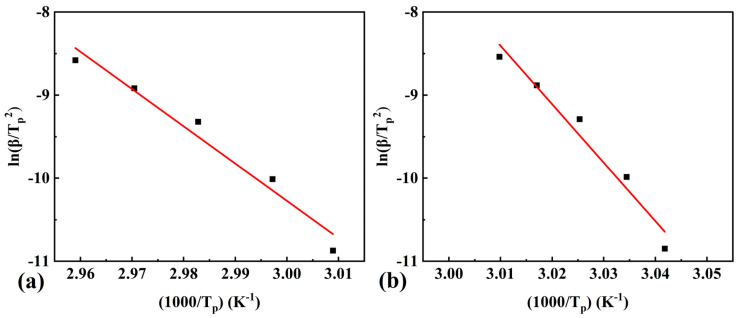
Fitting straight lines based on the Kissinger method corresponding to (**a**) pure OD and (**b**) OD/DMDBS/EG composite, the dots indicate data points with different linear heating rates for the Kissinger method, and the red lines indicate the straight lines of linear fit for the data points.

The Kissinger method (Equation (4)) was used to calculate the activation energy at peak temperatures corresponding to the maximum conversion rate (α bout 0.62 for pure OD and 0.84 for the composite PCM). The results obtained at several heating rates are presented in Figure 4a,b. Table 2 shows the values of activation energy, standard deviation, and correlation coefficient. The value of activation energy calculated using Kissinger’s method for the composite PCM was significantly higher than that of pure OD.

**Figure 5:**
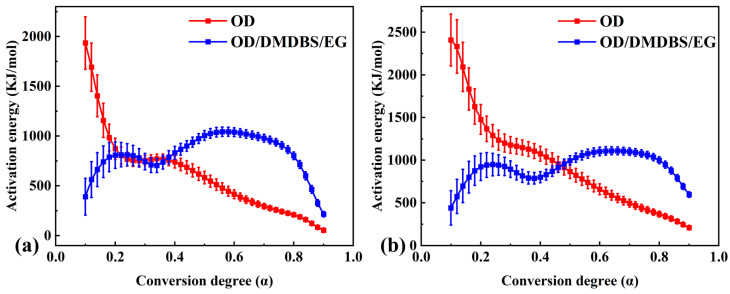
Dependence of activation energy on conversion degree for pure OD and OD/DMDBS/EG composite, calculated by using (**a**) the Friedman method and (**b**) the Ozawa–Flynn–Wall (OFW) method.

**Figure 6:**
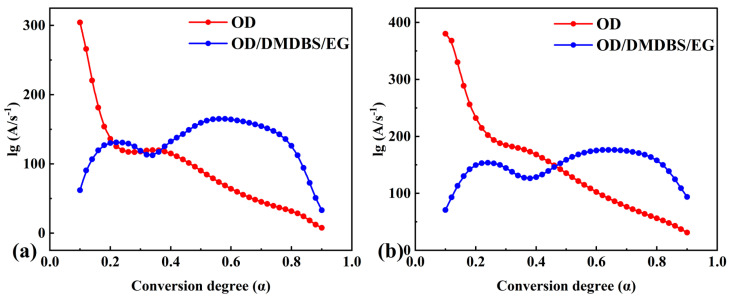
Dependence of pre-exponential factor on conversion degree for pure OD and OD/DMDBS/EG composite, calculated by using (**a**) the Friedman method and (**b**) the OFW method.

The conversion degree versus temperature curves and the conversion rate versus temperature curves were used to calculate the values of activation energy and the standard deviation of pure OD and the composite PCM phase transition processes using the isoconversional differential Friedman method (Equation (5)) and integral OFW method (Equation (6)) in the NETZSCH Thermokinetics 3.1 software (Figure 5). In the case of the Friedman method, for each given conversion degree, the plot of ln [β(dα/dT)] against 1/T obtained from the conversion rate versus temperature curves should be straight lines. The apparent activation could be determined from those slopes. In the case of the OFW method, the apparent activation could be determined from the slope plots of lnβ against 1/T obtained from the conversion degree versus temperature curves. In addition, the differential and integral forms of the mechanism function were assumed to be ƒ(α) = 1 − α and G(a) = ln(1 − a), respectively. The pre-exponential factor could be determined based on the corresponding intercept. The pre-exponential factor (logarithmic form) versus the conversion degree curves are shown in Figure 6.

As seen in Figure 5a,b, the strong dependency observed for the activation energy values on the conversion degree suggested that the phase transition processes of pure OD and the composite PCM may all pass through multiple steps [42]. Two activation energy versus conversion degree curves for pure OD show that the apparent activation energy calculated with both methods decreases with increasing conversion degree, and both curves have a flat step. For the composite PCM, an M-shaped profile (with two overlapping peaks) was observed in both activation energy versus conversion degree curves. Due to the addition of DMDBS and EG, at the initial stage of the phase transition process (α ≤ 0.2), the values of the activation energy of the composite PCM calculated with both methods were significantly lower than that of OD. However, at the latter stage of the phase transition process (α ≥ 0.5), the values of the activation energy of the composite PCM calculated with both methods were significantly higher than that of OD.

The values of activation energy calculated with the Friedman method for pure OD (α was 0.62, E = 360.02 KJ/mol) and the composite PCM (α was 0.84, E = 599.63 KJ/mol) were similar to those calculated with the Kissinger method. The agreement between the values of apparent activation energy obtained via the considered differential isoconversional methods (Kissinger and Friedman) shows that, in the investigated case, the different differential derivations used in these methods led to close results.

However, for a given α, the value of activation energy calculated using the Friedman method was almost always lower than that calculated using the OFW method. The reason for this difference was mainly due to the basic relation between the two methods [49,50]. In the case of a single-step reaction mechanism, which resulted in a weak dependence of the apparent activation energy on the conversion degree, the results calculated using the differential and integral methods were similar [37,51]. However, in the case of a complex reaction mechanism that led to a strong dependence of the apparent activation energy on the conversion degree, the results calculated using the differential and integral methods were significantly different [52,53,54]. The approximation of a constant apparent activation energy is the main source of error in the integral OFW method, whereas the Friedman method uses instantaneous rate values. For the prediction of reaction processes over a long time, the integral method may lead to inaccurate results. These explained the significant difference between the results calculated using the Friedman differential and OFW integration methods in this study.

Lastly, we note that some of the results calculated using the isoconversional method had comparatively high values of standard deviation. This may be due to the inherent errors of model-free methods in the presence of complex reaction mechanisms.

#### 3.2.2. Multivariate Non-Linear Regression Method

Then, the kinetic triplet (the apparent activation energy, the pre-exponential factor, and the most appropriate kinetic model) of the studied phase transition process was determined using the NETZSCH Thermokinetics 3.1 software. For simple reaction processes (single-step reactions), the choice was made to employ the multivariate linear regression method, and conversely, the multivariate non-linear regression method was required [42]. Friedman and OFW plots showed the dependence of the activation energy on the conversion degree, so the multivariate non-linear regression method was chosen. Due to the simultaneous consideration of DSC data at different linear heating rates, the accuracy of the applicable model was significantly improved using the multiple non-linear regression method compared to the single-curve method. Moreover, for complex reaction processes, it can directly fit experimental data with kinetic parameters to reduce errors [55].

The results from the isoconversional method were used as initial values and brought into a set of possible reaction models built into the software. A reaction model contained one or more reaction types. The reaction types and corresponding reaction equations in the NETZSCH Thermokinetics 3.1 software are shown in Table 3. These were then compared with the experimental data using the multiple non-linear regression method, and the kinetic parameters were optimized iteratively. Several reaction models were fitted using the multivariate non-linear regression method over a range of conversion degrees 0.05 ≤ α ≤ 0.95 and evaluated using statistical methods. The F-test was used to evaluate the most appropriate reaction model. Differences in the quality of fit between the various potential models were evaluated using the F-value (F_exp_) and the critical F-value at a confidence level of 0.95 (F_crit_(0.95)).

Table 4 shows the most appropriate kinetic models and kinetic parameters (the apparent activation energy and logarithmic form of the pre-exponential factor) for pure OD and the studied composite PCM. The phase transition processes for pure OD and the composite PCM could be satisfactorily described using a two-step consecutive reaction model (A → B → C). This corresponded to the two-step phase transition process described using the DSC endothermic curves (γ-phase → α-phase → liquid phase). According to the highest value of the correlation coefficient and the F_exp_ value (F_exp_ = 1.00 < F_crit_ (0.95) = 1.15), the first stage of the phase transition process for pure OD followed a three-dimensional diffusion (Jander’s type) reaction (D3), whereas the second stage was an n-dimensional nucleation growth process according to Avrami–Erofeev (An). For the composite PCM, the first stage of the phase transition process was an nth-order reaction (Fn), and the second stage, similar to that of pure OD, was an n-dimensional nucleation growth process according to Avrami–Erofeev (An).

In addition, similar to the results of the isoconversional method, the value of activation energy of the first step of the composite PCM was significantly decreased compared to that of pure OD due to the introduction of DMDBS and EG, while the value of the activation energy of the second step was significantly increased.

**Figure 7:**
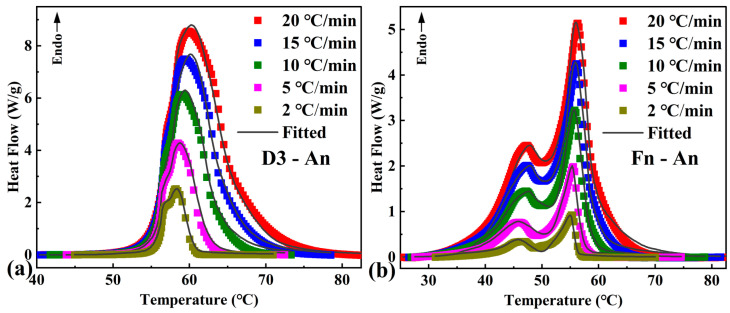
DSC endothermic curves and the corresponding fitting curves of (**a**) pure OD and (**b**) OD/DMDBS/EG composite at various linear heating rates.

For the determined kinetic parameters, the contribution of each step and linear heating rate β, the relationship between dα/dT and T, and the relationship between α and T can be obtained from the kinetic differential (Equation (2)) and integral (Equation (3)) equations for the phase transition process, respectively. According to Equation (1), α is defined by the enthalpy of the phase transition process, which the integral of the heat flow can determine, so the heat flow versus temperature curves can be obtained via software calculation. Figure 7a,b shows the results of fitting the calculated and experimental values for pure OD and the composite PCM at five different heating rates. Good agreement was shown between the most appropriate kinetic models obtained using the multiple nonlinear regression method and the experimental data.

**Figure 8:**
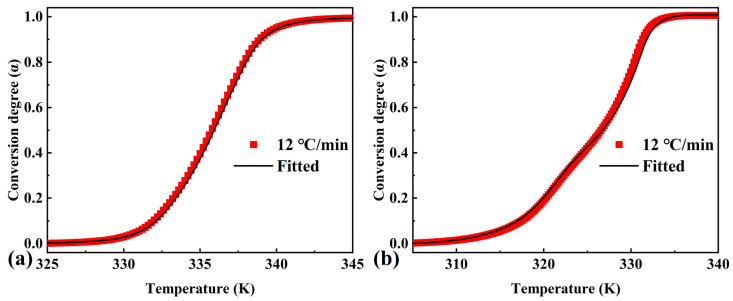
Checking the validity of the kinetic parameters, DSC endothermic curves and the corresponding fitting curves of (**a**) pure OD and (**b**) OD/DMDBS/EG composite at a linear heating rate of 12 °C/min.

The kinetic parameters listed in Table 4 were used to obtain conversion degree versus temperature curves corresponding to DSC data recorded at a heating rate of 12 °C/min that was not used in the kinetic analysis. The conversion degree versus temperature curves of both pure OD and the composite PCM were in good agreement with the results obtained directly from the DSC data (Figure 8). This was an argument for the validity of the obtained kinetic parameters of the phase transition processes.

### 3.3. Characterization and Discussion

**Figure 9:**
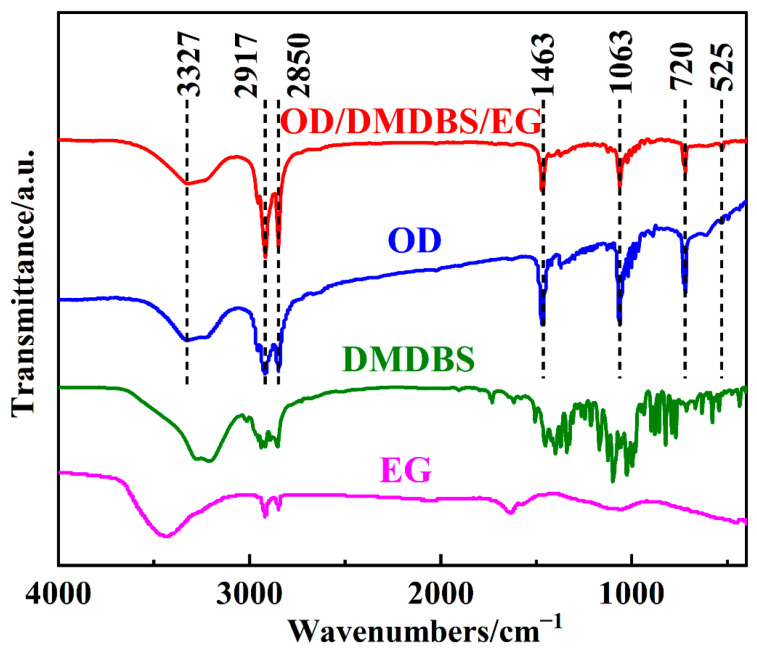
Fourier Transform Infrared (FTIR) spectrum of the samples.

Next, the composite PCM was characterized to discuss the changes during the phase transition process. Figure 9 shows the FTIR spectrum of pure materials and the composite PCM. Absorption bands of pure OD included the out-of-plane deformation, in-plane deformation, and stretching vibration of the O-H group (720 cm^−1^, 1463 cm^−1^, and 3327 cm^−1^, respectively); the C-H symmetrical and asymmetric stretching of the -CH_2_ group (2850 cm^−1^ and 2917 cm^−1^, respectively); the stretching vibration of C-O (1063 cm^−1^); and the vibration peak of the C-C skeleton (525 cm^−1^). As shown in the figure, the absorption peaks of the composite PCMs were not significantly different from those of pure OD. As a result, the chemical structure of the adsorbed OD did not change after compounding with the additives DMDBS and OD, and there was only physical force between the adsorbed OD and the additives.

**Figure 10:**
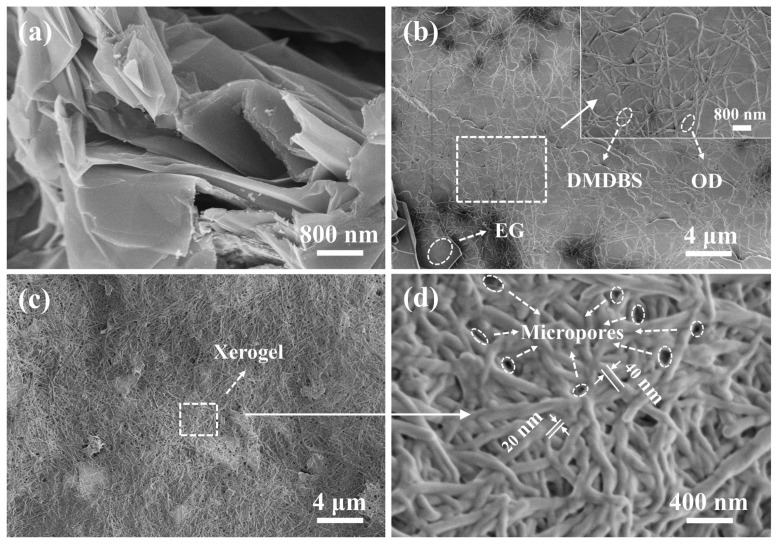
Scanning Electron Microscopy (SEM) micrographs of (**a**) EG, (**b**) OD/DMDBS/EG composite PCM, and (**c**,**d**) the xerogel.

The microscopic morphology of EG, the xerogel, and the composite PCM at different magnifications were observed using SEM. The xerogel was prepared as follows: the composite PCM was added to n-hexane and washed thoroughly, and the white precipitate of pure OD was filtered and then dried under vacuum for 24 h. EG had a distinct layer structure after expansion at high temperatures (Figure 10a). The graphite layers were interlaced with each other to form abundant micropores, which could support and adsorb the organic composites. From Figure 10b, it can be found that the surface of EG was coated with a large amount of OD/DMDBS gelatinous material, and the micropores were also filled. Moreover, OD was dispersed into small sizes and distributed in a fibrous network formed by DMDBS. When the ambient temperature was higher than the melting point of OD, both DMDBS and EG could adsorb OD and impede the movement of OD. After removing the OD, the EG was covered with a large number of xerogels, and a dense three-dimensional network formed through the self-assembly of DMDBS could be observed (Figure 10c). As shown in Figure 10d, the width of the fibers that make up the dense network was about 30 nm, and the size of the micropores for adsorbing OD in the interior of the network also reached the nanoscale. It could be assumed that the adsorbed OD was dispersed into extremely small sizes.

The size effect of the phase change material and the nanoconfinement effect of the three-dimensional network framework may be the main reasons why the adsorbed OD in the composite PCM exhibited a different phase change process from that of the free OD. Similar studies were conducted and explained by Masayuki et al. [56] and Huang et al. [57], respectively. The mass and size influenced crystal morphology and phase transition behavior. When the mass and size of OD were reduced to a certain level, both the solid-state transition temperature and the melting temperature decreased. The melting temperature was affected not only by the crystal/air surface but also by the crystal/substrate interface, resulting in a greater decrease in temperature for solid–solid phase transition than for solid–liquid phase transition [56]. The confinement of the three-dimensional network framework may also lead to an increase in the activation energy of the solid–liquid phase transition of the adsorbed OD. In addition, the three-dimensional network framework may affect the crystallization of the adsorbed OD. Due to the enlarged crystal unit cell of the OD, the phase transition process from γ-phase to α-phase requires lower energy, and the interval between solid–solid and solid–liquid phase transition processes increased [57]. These were in agreement with the results calculated and fitted using non-isothermal kinetics.

### 3.4. Kinetic Predictions

**Figure 11:**
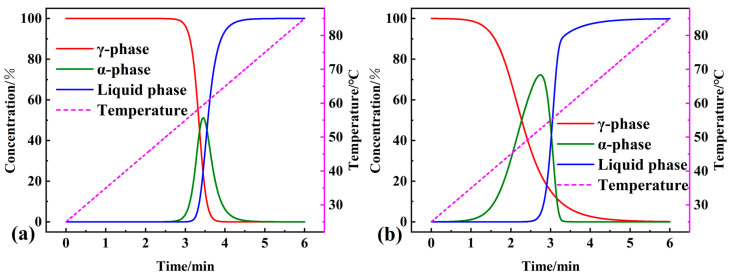
Results of the non-isothermal simulation of the phase transition process of (**a**) pure OD and (**b**) OD/DMDBS/EG composite.

The kinetic parameters and kinetic models obtained from the multiple nonlinear regression methods were used as starting parameters to predict and simulate the phase transition processes of pure OD and the studied composite PCM under several isothermal and non-isothermal conditions, respectively. The kinetic differential (Equation (2)) and integral equation (Equation (3)) for the phase transition process can be changed to dα/dt = Aexp [−E/R(T_0_ + βt)]ƒ (α) and G(α) = Aexp [−E/R(T_0_ + βt)]t (t denotes the linear heating time). For the determined kinetic parameters, the contribution of each step and linear heating rate β, and the relationship between conversion (each step) and time, can be obtained. Numerical simulations were carried out using NETZSCH Thermokinetics 3.1 software. The results of non-isothermal simulations at 10 K/min linear heating rates of the phase transition processes of pure OD and the composite PCM are shown in Figure 11a,b. The time interval between the first step and the second step of the reaction of the composite PCM was more significant, which was in agreement with the results of the DSC endothermic curves. In addition, the highest concentration of α-phase in the three phases during the phase transformation process of composite PCM reached 72.3%, which was higher than that of pure OD (51.1%).

**Figure 12:**
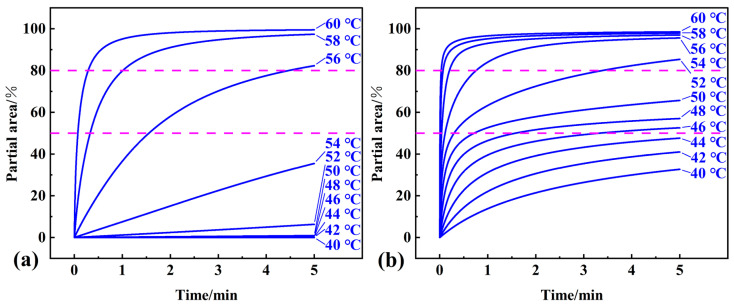
Results of the isothermal simulation of the phase transition process of (**a**) pure OD and (**b**) OD/DMDBS/EG composite in the temperature range of 40 °C–60 °C.

To evaluate the available temperature range of pure OD and composite PCM, isothermal simulations were performed in the temperature range of 40 °C–60 °C. For isothermal conditions, the kinetic differential and integral equation for the phase transition process can also be changed to dα/dt = Aexp(−E/RT)ƒ (α) and G (α) = Aexp(−E/RT)t. For the determined kinetic parameters and contribution of each step, the relationship between conversion and time can be obtained via the interpolation and extrapolation of the thermokinetics software. The conversion versus time curves with temperature as a parameter of pure OD and composite PCM are shown in Figure 12a,b. As shown in Figure 12, the conversion of the composite PCM reached 85.3% after 5 min at 52 °C, while the conversion of pure OD could not exceed 80% until the isothermal heating temperature reached 56 °C. After 5 min at 46 °C, the conversion of composite PCM reached 52.6%, while the conversion of pure OD was only 35.5% at 54 °C. Thus, the composite PCM had a wider available temperature range compared to pure OD.

**Figure 13:**
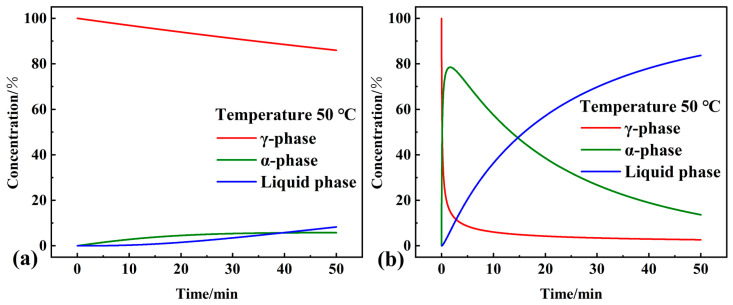
Results of the isothermal simulation of the phase transition process of (**a**) pure OD and (**b**) OD/DMDBS/EG composite after 50 min at 50 °C.

**Figure 14:**
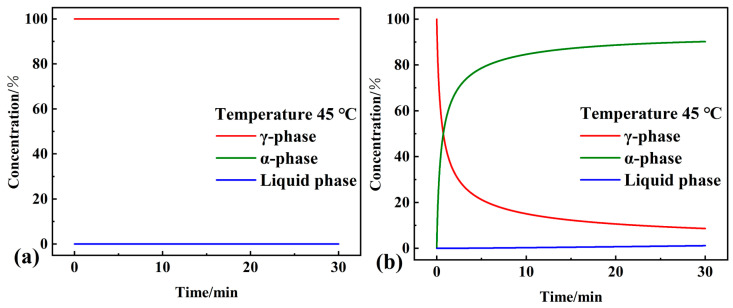
Results of the isothermal simulation of the phase transition process of (**a**) pure OD and (**b**) OD/DMDBS/EG composite after 30 min at 45 °C.

Then, the course of the concentration of each phase at a predetermined isothermal temperature was investigated. At a constant temperature, using the method for obtaining Figure 12, the concentration curves of each phase as a function of time can be obtained via the thermokinetics software calculations of the two steps of the phase transition process. As shown in Figure 13a,b, after 50 min at 50 °C, the concentration of the liquid phase of the composite PCM reached 83.7%, the concentration of the γ-phase was only 2.7%, and the phase transition process was close to the end. However, under the same conditions, neither the concentration of the liquid phase nor the α phase of pure OD exceeded 10%, and the concentration of the γ-phase was still 85.9%. In particular, as shown in Figure 14a,b, the concentration of the α phase of the composite PCM reached 90.2% after 30 min at 45 °C, and the concentration of the liquid phase was only 1.2%, with almost no liquid phase produced. The phase transition process of composite PCM was similar to the solid–solid phase transition process, which could avoid the leakage of PCM. Under the same conditions, almost no phase change occurred in pure OD.

## 4. Conclusions

The phase transition process of OD/DMDBS/EG composite PCM was investigated by analyzing the non-isothermal data at five linear heating rates using isoconversional and multivariate non-linear regression methods. The apparent activation energies were determined using both model-free isoconversional differential (Kissinger and Friedman) and integral (OFW) methods, respectively. The effect of DMDBS and EG resulted in a significant decrease in the activation energy of the composite PCM compared to pure OD at the initial stage (α ≤ 0.2) of the phase transition process and an increase at the later stage (α ≥ 0.5). Furthermore, the kinetic triplet state of the composite PCM was established using the multivariate non-linear regression method. The composite PCM (Fn-An) involved a two-step consecutive phase transition process but with a different reaction mechanism in the first step compared to pure OD (D3-An). The microscopic morphology of the composite PCM was analyzed, and it was found that the size effect and the nanoconfinement effect may be the main reasons for the increase in the interval between the two steps of the phase transition process and the change in the activation energy.

Finally, based on the obtained kinetic parameters, the phase transition process of the composite PCM was predicted under several isothermal and non-isothermal conditions. The predicted results showed that, below the peak temperature of pure OD, the composite PCM had a wider available temperature range than pure OD. Under specific conditions, the phase transition process of the composite PCM was similar to the solid–solid phase transition, which was effective in avoiding the leakage that is common in PCMs.

## Figures and Tables

**Table 1:** The peak temperatures and the melting enthalpies of pure OD and OD/DMDBS/EG composite PCM at several linear heating rates.

Samples	T_p_ (°C)					ΔH_m_ (J/g)				
	2 °C/min	5 °C/min	10 °C/min	15 °C/min	20 °C/min	2 °C/min	5 °C/min	10 °C/min	15 °C/min	20 °C/min
OD	59.2	60.5	62.1	63.5	64.8	245.5	246.3	246.1	245.3	244.9
OD/DMDBS/EG	55.6	56.4	57.4	58.3	59.1	195.4	196.5	197.3	196.1	195.9

**Table 2:** Values of activation energy, standard deviation, and correlation coefficient calculated with the Kissinger method.

Sample	Activation Energy, E (KJ/mol)	Correlation Coefficient
OD	373.138 ± 40.198	0.983
OD/DMDBS/EG	584.905 ± 64.550	0.982

**Table 3:** Reaction types and corresponding reaction equations in the NETZSCH Thermokinetics 3.1 software.

Code	ƒ(α)	Reaction Type
F1	1 − α	first-order reaction
F2	(1 − α)^2^	second-order reaction
Fn	(1 − α)^n^	nth-order reaction
R2	2·(1 − α)^1/2^	two-dimensional phase boundary reaction
R3	3·(1 − α)^2/3^	three-dimensional phase boundary reaction
D1	1/2α	one-dimensional diffusion
D2	−1/ln(1 − α)	two-dimensional diffusion
D3	1.5·(1 − α)^1/3^[(1 − α)^−1/3^ − 1)	three-dimensional diffusion (Jander’s type)
D4	1.5/[(1 − α)^−1/3^ − 1]	three-dimensional diffusion (Ginstling–Brounstein type)
B1	α·(1 − α)	simple Prout–Tompkins equation
Bna	α^a^·(1 − α)^n^	expanded Prout–Tompkins equation (na)
C1-X	(1 − α)·(1 + Kcat·X)	first-order reaction with autocatalysis through the reactands, X X = a product in the complex model, frequently X = α.
Cn-X	(1 − α)^n^(1 + Kcat·X)	nth-order reaction with autocatalysis through the reactands, X
A2	2·(1 − α)[−ln(1 − α)]^1/2^	two-dimensional nucleation
A3	3·(1 − α)[−ln(1 − α)]^2/3^	three-dimensional nucleation
An	n·(1 − α)[−ln(1 − α)]^(n−1)/n^	n-dimensional nucleation/nucleus growth according to Avrami/Erofeev

**Table 4:** Kinetic parameters calculated using the multiple non-linear regression method for pure OD and OD/DMDBS/EG composite.

Sample	Kinetic Parameters		
OD	1st Step	2nd Step 2	
	Mechanism, D3	Mechanism, An	F_exp_ = 1.00
	Activation energy, E (KJ/mol) = 953.895	Activation energy, E (KJ/mol) = 408.089	F_crit_ = 1.15
	Pre-exponential factor, lg(A/s^−1^) = 149.616	Pre-exponential factor, lg(A/s^−1^) = 53.135	Correlation coefficient, 0.993
		Dimension, n = 1.895	
OD/DMDBS/EG	1st Step 1	2nd Step 2	
	Mechanism, Fn	Mechanism, An	F_exp_ = 1.00
	Activation energy, E (KJ/mol) = 306.872	Activation energy, E (KJ/mol) = 844.761	F_crit_ = 1.13
	Pre-exponential factor, lg(A/s^−1^) = 48.905	Pre-exponential factor, lg(A/s^−1^) = 133.475	Correlation
	Reaction order, n = 2.947	Dimension, n = 0.798	coefficient, 0.995

## Data Availability

The data in this manuscript are available on request.
